# Insurance Status and Income Proxies Are the Most Consistent Predictors of Disparities in Access to Care and Outcomes After Medial Patellofemoral Ligament Reconstruction in the United States: A Systematic Review

**DOI:** 10.1016/j.asmr.2025.101268

**Published:** 2025-09-25

**Authors:** Erin L. Brown, Kenneth T. Nguyen, Daman P. Dhunna, Laura A. Wright, Shreya M. Saraf, Saijayanth Mosalakanti, Mary K. Mulcahey

**Affiliations:** a Tulane University School of Medicine, New Orleans, Louisiana, U.S.A.; b Chobanian & Avedisian School of Medicine, Boston University, Boston, Massachusetts, U.S.A.; c Rudolph Matas Library of the Health Sciences, Tulane University School of Medicine, New Orleans, Louisiana, U.S.A.; d Department of Orthopaedic Surgery and Rehabilitation, Loyola University Medical Center, Maywood, Illinois, U.S.A.

## Abstract

**Purpose:**

To evaluate the influence of health care access, insurance coverage, racial and ethnic identity, income-related proxies, employment status, preventive care use, and geographic location on the diagnosis, treatment, and outcomes after medial patellofemoral ligament reconstruction (MPFLR).

**Methods:**

A systematic review of electronic databases was performed according to the Preferred Reporting Items for Systematic Reviews and Meta-Analyses guidelines to identify Level II-IV clinical studies related to patellar instability that were published between January 2010 and January 2025. Studies were included if they were peer-reviewed English-language studies detailing the socioeconomic and demographic factors of outcomes after MPFLR. Case reports, systematic reviews, animal and cadaver studies, and publication formats other than peer-reviewed journal studies were excluded.

**Results:**

Seven studies met inclusion criteria, which resulted in a total 983,985 patients (983,658 female, 99.97%, and 327 male, 0.03%). Three studies (42.9%) found that insurance status affected the evaluation and treatment of patients with patellar instability, and subsequent outcomes after MPFLR, with those who were privately insured experiencing a better clinical course with regards to evaluation, treatment, and outcomes. Four (57.1%) studies identified race or ethnicity as a factor that influenced the prevalence of patellar instability, treatment, cost, and outcomes. One study (12.5%) found certain markers of high income such as home ownership, full-time employment, and having a recent health check-up positively affected the evaluation, treatment, and postoperative outcomes of patients with patellar instability.

**Conclusions:**

This systematic review found that lower socioeconomic status, public insurance coverage (as opposed to private coverage), and minority racial or ethnic identity were associated with delayed evaluation, lower likelihood of surgical intervention, and reduced postoperative compliance or satisfaction among patients undergoing MPFLR. Patients with these characteristics experience longer wait times from injury to clinic evaluation and have reduced odds in selection as a candidate for surgery. Postoperative satisfaction was also markedly worse for surgical patients with these characteristics.

**Level of Evidence:**

Level IV, systematic review of Level III and IV studies.

The primary factor responsible for preventing lateral movement of the patella is the medial patellofemoral ligament (MPFL), which is frequently torn when the patella is dislocated.^1^ Untreated recurrent lateral patellar instability has been associated with intra-articular cartilage damage that may impact knee function in both daily and athletic activities.^1^ Medial patellofemoral ligament reconstruction (MPFLR) is one of the most common surgical treatments for patellar instability.^2^ Socioeconomic factors (income, level of education, insurance status, etc.) may impact the early recognition of patellar instability and the ability to pursue appropriate treatment.^2^

Disparities in health care outcomes manifest in various forms, including differences in access to care, insurance coverage, quality and timeliness of treatment, and geographic availability of specialized services.^2^ Understanding the underlying cause of these disparities is essential to develop effective strategies for eliminating them. In investigating the influence of these disparities, we operationally define social determinants of health as health care access, insurance coverage, racial and ethnic identity, income-related proxies, employment status, preventive care use, and geographic location. These measures of disparities play a significant role in the health care disparities that patients experience when undergoing orthopaedic procedures, including MPFLR.^3^ Previous studies have found that socioeconomic and patient-related factors such as age, sex, socioeconomic status, and race impact the odds of undergoing many orthopaedic surgeries (eg, anterior cruciate ligament reconstruction, total joint arthroplasty, and spine surgery) as well as the associated outcomes.^4^, ^5^, ^6^ Identifying the socioeconomic and demographic factors that influence care for patients with patellar instability may enable orthopaedic surgeons to intervene at an earlier stage in the disease process and potentially lead to more favorable postoperative outcomes.

The few studies published regarding the socioeconomic factors that influence treatment of patellar instability have found that race, sex, and insurance status may be significant predictors of access to surgical care, timing of initial evaluation and surgery, and cost of treatment.^7^^,^^8^ In 2023, Shankar et al.^9^ performed a single-institution retrospective case study investigating socioeconomic predictors of time to initial evaluation, time to surgery, and postoperative outcomes among patients with lateral patellar instability undergoing MPFLR. The authors found that markers of greater socioeconomic status, including having a general check-up in the year before surgery, home ownership, and full-time employment were predictive of shorter time to initial evaluation, shorter time to surgery, and superior postoperative outcomes after MPFLR. In addition, previous studies have reported that patient sex is a social determinant of health that impacts outcomes after MPFLR. Female patients have a 5.45 times greater risk of any postoperative complications; greater 1-, 5-, and 8-year risk of recurrent dislocation or instability, and worse postoperative outcome scores.^10^, ^11^, ^12^ Associations between several socioeconomic factors, treatment delays, and poor postoperative outcomes are well-documented in the literature for procedures including but not limited to shoulder arthroplasty and anterior cruciate ligament reconstruction.^13^, ^14^, ^15^, ^16^ However, there is a paucity of literature regarding the impact of socioeconomic and demographic factors on outcomes after MPFLR. The purpose of this study was to evaluate the influence of health care access, insurance coverage, racial and ethnic identity, income-related proxies, employment status, preventive care use, and geographic location on the diagnosis, treatment, and outcomes after MPFLR. We hypothesized that health care access, insurance coverage, racial and ethnic identity, income-related proxies, employment status, preventive care use, and geographic location would significantly influence treatment and outcomes in patients with patellar instability.

## Methods

### Search Strategy

A systematic review of PubMed, Web of Science, Embase, and CINAHL was performed according to the Preferred Reporting Items for Systematic Reviews and Meta-Analyses in January 2025.^17^ The primary socioeconomic or demographic factors of interest were health care access, insurance coverage, and racial and ethnic identity. Additional factors of interest not explicitly searched for were income-related proxies, employment status, preventive care use, and geographic location. Search terms included “medial patellofemoral ligament reconstruction,” “patellar instability,” “socioeconomic factors,” “insurance,” “race,” and “health services accessibility.” Studies published between January 2010 and January 2025 that were conducted in the United States were taken into consideration for inclusion. Appendix Table 1, available at www.arthroscopyjournal.org, contains a complete documentation of the database search strategies.

### Eligibility Criteria

Peer-reviewed English-language studies examining at least 1 socioeconomic or demographic factor impacting outcomes after MPFLR were included. Studies were excluded if they were case reports, systematic reviews, animal and cadaver studies, and publication formats other than peer-reviewed journal articles.

### Study Selection Criteria and Procedures

The Covidence systematic review program was used to upload all identified studies. Independently and collaboratively, 2 reviewers (E.L.B. and K.T.N.) assessed the eligibility of studies that examined socioeconomic and demographic factors affecting outcomes after MPFLR. After full-text evaluation, studies that met inclusion criteria were included in this systematic review. Disputes regarding selection were settled by consensus. This was implemented to improve the protocol and data inclusion, strengthen the objectivity, and prevent errors in the research selected for the study. The Risk Of Bias in Non-Randomized Studies – of Interventions (ROBINS-I) tool was used to evaluate the risk of bias in observational studies, cross-sectional research, systematic reviews, and nonrandomized trials.^18^

### Data Extraction

The following data were extracted from all studies that met inclusion criteria: race/ethnicity, insurance, household income, socioeconomic status, as well as validated outcome scores such as visual analog scale (VAS) satisfaction and the Kujala score.

## Results

**Fig 1:**
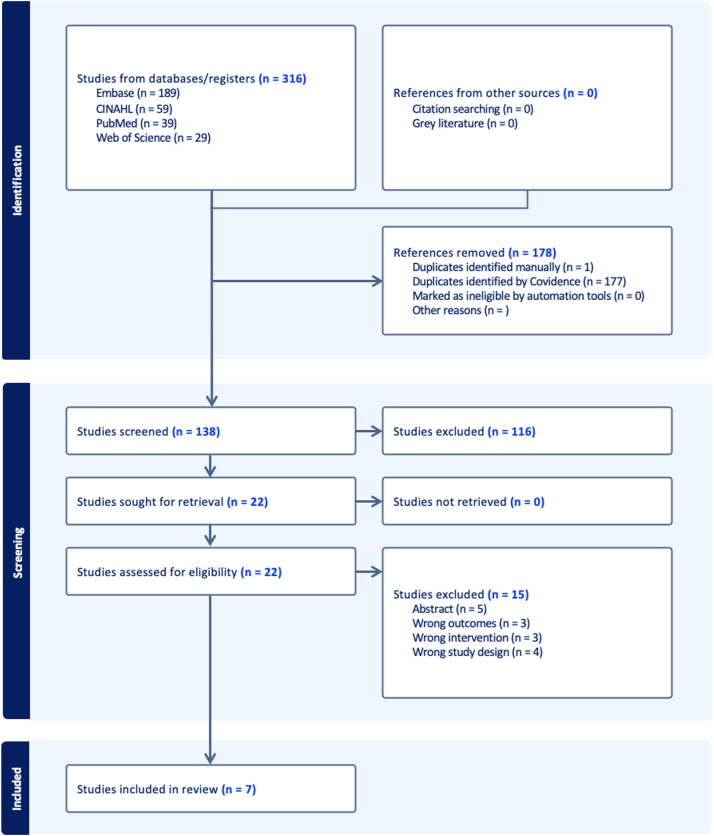
Preferred Reporting Items for Systematic Reviews and Meta-Analysis (PRISMA) flow diagram.

The initial search identified 316 studies. After eliminating 178 duplicates, the 2 authors screened 138 abstracts and titles. After this, 22 studies underwent a full-text review. Seven studies met inclusion criteria with a total of 983,985 patients (983,658 female, 99.97%, and 327 male, 0.03%) (Fig 1, Table 1).^7^, ^8^, ^9^, ^10^^,^^19^, ^20^, ^21^

**Table 1:** Characteristics of Studies Included in the Systematic Review

Study	Study Design	Level of Evidence	Number of Participants	Patient Sex	Follow-Up Time	Barrier(s) Identified
Allahabadi et al, 2022^7^	Retrospective case series	III	78	46 female, 32 male	601 ± 418 d	Sex, age, race/ethnicity, insurance status
Li et al., 2021^8^	Retrospective cohort	III	2,557	1,384 female, 1,173 male	Not explicitly stated	Sex, age, race/ethnicity, insurance status
Shankar et al., 2023^9^	Retrospective case series	IV	70	51 female, 19 male	45.7 ± 25.3 mo	Sex, age, race/ethnicity, insurance, markers of high income
Hiemstra and Kerslake, 2019^10^	Cohort	III	298	225 female, 73 male	26.4 mo	Sex
Howard et al., 2024^19^	Retrospective cohort	III	85	48 female, 37 male	4-7 mo	Race/ethnicity, socioeconomic status
Walawski et al., 2024^20^	Prospective cohort	III	39	26 female, 13 male	Both 6 mo and 12 mo	Geographic, rural vs urban locations
Martinazzi et al, 2023^21^	Cross-sectional study	III	980,878	980,878 female	Not explicitly stated	Sex, contraceptive use

### Risk of Bias Assessment

The ROBINS-I tool was used to evaluate the risk of bias in eligible studies (Table 2). Most studies reported strong methodologic quality and had low concern for bias attributable to the selection of participants, classification of interventions, deviations from intended interventions, missing data, measurement outcomes, and in the selection of the reported results. Thus, all of the studies only had low-to-moderate concerns for overall bias.

**Table 2:** Quality and Risk of Bias Assessment Using the Risk Of Bias in Non-Randomized Studies – of Interventions (ROBINS-I) Tool

Study	Confounding Bias	Participant Selection Bias	Intervention Classification Bias	Bias due to Deviations From Intended Interventions	Bias due to Missing Data	Bias in Measurement Outcomes	Bias in Selection of the Reported Results	Overall Bias
Allahabadi et al., 2022^7^	Moderate	Low	Low	Low	Low	Low	Moderate	Moderate
Li et al., 2021^8^	Low	Low	Low	Low	Low	Moderate	Low	Low
Shankar et al., 2023^9^	Moderate	Low	Low	Low	Low	Moderate	Low	Moderate
Hiemstra and Kerslake, 2019^10^	Low	Low	Low	Low	Low	Low	Low	Low
Martinazzi et al., 2023^21^	Low	Low	Low	Low	Low	Low	Low	Low
Walawski et al., 2024^20^	Low	Low	Low	Low	Moderate	Low	Low	Moderate
Howard et al., 2024^19^	Low	Low	Low	Low	Moderate	Low	Low	Low

### Insurance Status

Three of 7 studies (42.9%) evaluated the impact of insurance status on the evaluation and treatment of patients with patellar instability, and subsequent outcomes after MPFLR. Across the 4 studies examining the impact of insurance status, it was found that patients with commercial insurance made up the majority of admissions for patellar instability. Table 3 provides a summary of the cited studies, their study population, the insurance-related findings, and key takeaways.

**Table 3:** Summary of Insurance-Related Disparities in Patient Outcomes After MPFL Reconstruction

Study	Population and Context	Insurance-Related Findings	Key Takeaway
Shankar et al., 2023^9^	Retrospective case series at single institution; N = 70	65 of 70 (92.9%) had private/commercial insurance.	Insurance status in combination with other factors such as full-time employment and having a general health check-up within the year prior was associated with outcomes and patient satisfaction.
Li et al., 2021^8^	NY State Statewide Planning and Research Cooperative System (SPARCS) database (2016–2018); N = 2,557 patients ≤21 years	Surgery rate: uninsured = 0% (*P* = .009); privately insured = 5.5%. emergency department (ED) use: uninsured more likely than private (odds ratio 1.881, *P* = .016). Medicaid less likely than private to use ED (odds ratio 0.640, *P* < .001).	Patients without insurance were more likely than patients with private insurance to present to the ED for initial care of patellar instability (odds ratio 1.881, *P* = .016).
Allahabadi et al., 2022^7^	Safety-net tertiary center, pediatric/adolescent population; N = 78	Public insurance associated with: longer time to clinic (466 vs 77 days, *P* = .002), magnetic resonance imaging (466 vs 82 days, *P* = .003), surgery (695 vs 153 days, *P* = .0003), and evaluation-to-surgery delay (226 vs 73 days, *P* = .002). No difference in intraoperative metrics.	Publicly insured patients had increased time from initial injury to clinical evaluation as well as a longer wait time from clinical evaluation to surgery.

In 2023, Shankar et al.^9^ performed a retrospective case series to identify socioeconomic predictors (e.g., insurance status) of time to initial evaluation, time to surgery, and postoperative outcomes among patients with patellar instability undergoing MPFLR with allograft. The authors found that insurance status in combination with other factors such as full-time employment and having a general health check-up within the year prior was associated with outcomes and patient satisfaction.^9^

Li et al.^8^ performed a retrospective review of the New York Statewide Planning and Research Cooperative System (SPARCS) database from 2016 to 2018 to identify patients aged 21 and younger who were diagnosed with patellar instability. The authors found that patients without insurance did not undergo surgery (surgery rate of 0%; *P* = .009), whereas privately insured patients had a surgery rate of 5.5%. The authors also noted that patients without insurance were more likely than patients with private insurance to present to the emergency department (ED) for initial care of patellar instability (odds ratio [OR] 1.881, *P* = .016). Compared with privately insured patients, patients with Medicaid were less likely to use the ED for this purpose (OR .640, *P* < .001).^8^

In 2022, Allahabadi et al.^7^ conducted a retrospective cohort study at a pediatric and adolescent safety-net tertiary referral center in a large metropolitan region, focusing on patients who underwent MPFL repair or reconstruction. The authors found that compared with the private insurance group, patients with public insurance were older (16.1 ± 2.7 years vs 14.6 ± 1.8 years, *P* = .019), comprised a lower proportion of White patients (15.8% vs 52.5%; *P* = .0005), comprised a greater proportion of Hispanic ethnicity (55.3% vs 15.0%; *P* = .0001), and comprised a greater proportion of Spanish-speaking patients (21.1% vs 2.5%; *P* = .007). In addition, publicly insured patients had increased time from initial injury to clinical evaluation (466 vs 77 days; *P* = .002), magnetic resonance imaging (MRI) (466 vs 82 days; *P* = .003), and surgery (695 vs 153 days; *P* = .0003), as well as a longer wait time from clinical evaluation to surgery (226 vs 73 days; *P* = .002). Conversely, no differences between public and private insurance were identified in terms of degree of pathology (as measured by preoperative radiographic or MRI-based evaluation of anatomic factors). Similarly, there was no difference in insurance status regarding the number of patients requiring concomitant procedures at the time of MPFLR (68.4% vs 62.5%; *P* = .58), tourniquet time (61.4 vs 60.3 minutes; *P* = .85), or in the proportion of patients undergoing MPFL repair or reconstruction.^7^

### Race/Ethnicity

Four of 7 (57.1%) studies identified race or ethnicity as a factor that influenced the prevalence, treatment, cost, and outcomes in patients with patellar instability (Table 4). Li et al.^8^ found that compared with White patients, Black patients had 0.428 times the odds of undergoing surgery for patellar instability (*P* = .004). Hispanic patients had 0.268 times the odds of surgery compared with White patients, but this difference was not significant (*P* = .069). In addition, Black patients had $566 more in charges than White patients (*P* = .009). The authors also found that White patients were the most likely to use the ED for initial care of patellar instability, whereas Black patients (OR .371, *P* < .001), and patients of another race (OR .320, *P* < .001) were significantly less likely to present to the ED for initial care.^8^

**Table 4:** Summary of Race/Ethnicity-Related Disparities in Patient Outcomes After MPFL Reconstruction

Study	Population and Context	Race/Ethnicity-Related Findings	Key Takeaway
Li et al., 2021^8^	NY State Statewide Planning and Research Cooperative System (SPARCS) database (2016-2018); N = 2,557 patients <21 years	Compared with White patients, Black patients had 0.428 times the odds of undergoing surgery for patellar instability (*P* = .004). Hispanic patients had 0.268 times the odds of surgery compared with White patients. In addition, Black patients had $566 more in charges than White patients (*P* = .009).	Patients without insurance were more likely than patients with private insurance to present to the emergency department (ED) for initial care of patellar instability (odds ratio 1.881, *P* = .016).
Allahabadi et al,. 2022^7^	Safety-net tertiary center, pediatric/adolescent population; N = 78	Surgery rate: uninsured = 0% (*P* = .009); privately insured = 5.5%. ED use: uninsured more likely than private (odds ratio [OR] 1.881, *P* = .016). Medicaid less likely than private to use ED (OR 0.640, *P* < .001).	Patients without insurance were more likely than patients with private insurance to present to the ED for initial care of patellar instability (OR 1.881, *P* = .016).
Howard et al., 2024^19^	Single-institution study; Hispanic adolescent population; N = 85	Hispanic patients in lower-income categories (<100% of state median income) had the lowest rate of physical therapy appointments attended (*P* = .044), whereas those in higher-income categories (>100% of state median income) had the greatest rate of postoperative clinic attendance (*P* = .019).	Hispanic patients in lower-income categories were significantly more likely to have public insurance (82.9%, as compared with 44.4%, *P* = .004).
Shankar et al., 2023^9^	Retrospective case series at single institution; N = 70	48 of 70 (68.6%) patients who underwent medial patellofemoral ligament reconstruction with allograft for treatment of lateral patellar instability were White, 7 (10.0%) were Black, 6 (8.6%) were Asian, 1 (1.4%) was Native American, American Indian, or Alaska Native, 1 (1.4%) was Native Hawaiian or other Pacific Islander, and 7 (10.0%) were other non-White races.	Insurance status in combination with other factors such as full-time employment and having a general health check-up within the year prior was associated with outcomes and patient satisfaction (*P* = .005).

Allahabadi et al.^7^ found that race, ethnicity, and primary language were statistically associated with insurance type (*P* < .0005), which ultimately impacted time to clinical evaluation, MRI obtainment, and surgery for patellar instability. Patients with private insurance tended to be White or Asian, whereas those with public insurance were more frequently Black. Those who had private insurance more commonly identified as Non-Hispanic and English speaking, whereas those who had public insurance were significantly more likely to identify as Hispanic- and Spanish-speaking.^7^

In addition, Howard et al.^19^ noted that being Hispanic alone was not associated with poorer postoperative outcomes. Instead, socioeconomic status had a synergistic effect with ethnicity toward compliance with postoperative care.^19^ The authors also emphasized that the median household income of Hispanic patients was significantly lower than non-Hispanic patients (*P* = .027). Although no significant differences were observed in the duration of follow-up or final Kujala scores between ethnic groups, disparities were primarily noted in rehabilitation attendance and follow-up compliance, which were strongly linked to income levels.^19^

In the study by Shankar et al.,^9^ race was found to be both a positive and negative predictor of postoperative satisfaction in interaction with other socioeconomic markers including level of college education, home ownership, and out-of-town vacation time. Non-White race was not an independent predictor of VAS satisfaction (*P* > .05). However, the interactions of non-White race with (1) having at least 2 years of college education and (2) taking at least 1 vacation in the year before surgery were predictive of lower VAS satisfaction (*P* < .05). In contrast, the interaction of non-White race with home ownership was predictive of greater VAS satisfaction (β = 42.6, *P* < .001).^9^

### Impact of Geographic Location

One study (12.5%), Walawski et al.,^20^ compared clinical and functional outcomes of MPFLR between rural and urban populations. Despite equivalent access to surgical care, patients in rural areas had significantly lower functional outcomes postoperatively compared with their urban counterparts. The study attributed these disparities to differences in access to high-quality rehabilitation services. Patients from rural areas often had to travel greater distances to reach rehabilitation centers, leading to less-frequent and consistent participation in physical therapy. Both groups showed similar improvements in clinical scores like Knee injury and Osteoarthritis Outcome Score and Kujala, but urban patients achieved better results in strength recovery and functional tests.^20^ The authors highlighted that although surgical techniques were standardized, unequal access to postoperative care perpetuated disparities in recovery outcomes between rural and urban settings.^20^

### Markers of High Income

One of the 7 studies (12.5%), Shankar et al.,^9^ found certain markers of high income to affect the time to evaluation, treatment, and postoperative outcomes for patients with patellar instability. Having a general health check-up in the year before surgery was predictive of shorter time to initial evaluation (β = − 100.5 [− 174.5, − 26.5 (95% CI)], *P* = .008).^9^ In addition, home ownership was predictive of a shorter time to surgery (β = − 56.5 [− 104.7, 8.3], *P* = .02). Lastly, full-time employment was predictive of greater VAS satisfaction (β = 14.1 [4.3, 23.9], *P* = .006) and greater Kujala score (β = 8.7 [0.9, 16.5], *P* = .03).^9^

### Sex

Although sex was not a primary search term in our systematic review, 5 studies (71.4%) described patient sex as a characteristic that affected the prevalence of patellar instability, with females being disproportionately affected. However, treatment with MPFLR and outcomes did not vary significantly by sex.

In the study by Allahabadi et al.,^7^ 46 of 78 (58.9%) patients with symptomatic patellar instability who underwent either MPFL repair or reconstruction were female. The authors found that patient sex did not have a statistically significant effect on insurance type (*P* = .967). Similarly, Shankar et al.^9^ found that 51 of 70 (72.9%) patients were female in their retrospective case series that enrolled patients who underwent MPFLR with allograft. Li et al.^8^ included 2,557 pediatric patients who sustained a patella dislocation or subluxation, most of whom were female (1,384, 54.1%). The authors found that the proportion of patients undergoing surgery after a patellar instability event, as opposed to nonoperative treatment, did not vary significantly by patient sex (*P* = .426). In addition, sex was not a predictor of odds of surgery after a patellar instability event; however, female patients were less likely to use the ED for initial care for patellar instability than male patients (odds ratio 0.691, *P* = .001).^8^

Hiemstra and Kerslake^10^ evaluated 328 patients with recurrent lateral patellar instability who underwent primary patellofemoral stabilization, 225 of whom were female (75.5%) and 73 were male (24.5%). When stratified by sex, baseline characteristics were not statistically different, except for older age at first dislocation for male patients (*P* = .022). The authors found that sex was not related to outcomes after MPFLR as assessed by the 12- and 24-month Banff Patellofemoral Instability Instrument scores.^10^

Martinazzi et al.^21^ performed a cross-sectional study of female patients aged 15 to 26 years who underwent various reconstruction procedures, with most cases being MPFLR, for patellar instability between 2012 and 2022. The authors found that female patients prescribed systemic contraceptives containing estrogen or progesterone had an increased rate of MPFLR. After 1-to-1 propensity score matching, 0.054% (525/980,878) of female patients prescribed an oral contraceptive containing ethinyl estradiol underwent reconstruction procedures for patellar instability compared with 0.043% (417/980,878) of controls (Relative Risk 1.3; 95% confidence interval 1.1-1.4; *P* = .0004).

### Age

Three studies (42.9%) evaluated age as a demographic factor that affected the time to evaluation and postoperative outcomes of patients with patellar instability. In 2019, Hiemstra and Kerslake^10^ reported older age at first patella dislocation for male patients (*P* = .022). Allahabadi et al.^7^ found that age was an independent predictor of the amount of time between patellar dislocation and being seen in clinic, such that older age was associated with a longer time from injury to clinic (*P* = .0446). However, age alone was not a predictor of odds of surgery after a patellar instability event.^7^

## Discussion

Across the included studies, insurance status and socioeconomic markers were the most consistent predictors of disparities in access to care and outcomes after MPFLR. The findings of this study indicate that patients with public or government insurance experience longer disease courses and poorer outcomes after MPFLR. Racial minorities are similarly affected, facing longer disease courses, and suboptimal outcomes. Markers of higher income, such as home ownership and full-time employment, are positively associated with shorter times to evaluation, treatment, and outcomes after MPFLR. In addition, patient sex and age were consistently found to impact the prevalence and disease course of patellar instability. Older age at the time of MPFLR was linked to poorer outcomes and delays in treatment. These results underscore the importance of addressing socioeconomic to improve patient care and outcomes after MPFLR.

Insurance status and type are important factors influencing the outcomes after MPFLR, because they have an impact on timely access to care. This issue was highlighted in more than one half of the studies analyzed, and the results align with the increasing body of data in the orthopaedic literature on disparities resulting from unequal access to care. Alvarez et al.^4^ found that insurance status may represent an independent prognosticator for outcomes after total joint arthroplasty. In addition, we noted that patients with public/government insurance had worse treatment access/use and outcomes. For patients undergoing MPFLR, delays in clinical evaluation and surgery, as well as decreased postoperative satisfaction were commonly associated with uninsured or non-commercial insurance status. This was emphasized by multiple studies.^7^, ^8^, ^9^^,^^21^

Socioeconomic status significantly impacts access to orthopaedic care and surgical outcomes. Our findings align with Patel et al.,^15^ who investigated the impact of socioeconomic factors on access to pediatric anterior cruciate ligament reconstruction. The authors found that publicly insured pediatric patients faced longer delays in evaluation, imaging, and surgery, leading to worse postoperative outcomes. Similarly, Nordenvall et al.^22^ found that patients with higher income and education levels were more likely to undergo surgical treatment for cruciate ligament injuries, highlighting socioeconomic disparities in orthopaedic care. Our findings suggest that financial and social factors directly influence treatment access and outcomes after MPFL reconstruction. Li et al.^8^ further suggest that racial disparities affect surgical decision-making, with White patients more likely to undergo MPFLR. These disparities underscore systemic barriers in orthopaedic care, emphasizing the need for policies that improve access, reduce financial obstacles, and promote equitable treatment for all patients.

Several studies have investigated the impact of sex and age on outcomes after MPFLR, with varying conclusions regarding their influence on treatment success. Our study observed that although men tend to undergo surgery at an older age, sex alone did not significantly impact postoperative outcomes. Instead, increasing age at the time of surgery was associated with poorer patient-reported outcomes. These findings align with Hiemstra and Kerslake,^10^ who specifically analyzed the relationship between age, sex, and MPFLR outcomes and concluded that age at the time of surgery—not sex—was the primary factor influencing postoperative quality of life. In contrast, other studies have reported significant sex-based disparities in MPFLR outcomes. Fancher et al.^23^ conducted a systematic review to assess sex-specific postoperative results and found that female patients had greater complication rates, greater recurrence of instability, and worse functional outcomes than male patients. Similarly, Figueroa et al.^24^ focused on sex-specific considerations in patellar instability and noted that female patients experienced worse outcomes, likely attributable to anatomical differences such as trochlear dysplasia, patella alta, and ligamentous laxity.

Several studies in our review suggest an association between age at the time of MPFLR and postoperative outcomes; however, data in the literature are not sufficient to confirm this as a definitive predictor. Nonetheless, our findings reinforce the understanding that age at surgery is a critical predictor of outcomes after MPFLR, and challenges the notion that sex alone dictates poorer postoperative results. The observed trend suggests that an individualized surgical approach accounting for anatomical and hormonal factors may be more relevant in optimizing outcomes. For example, some anatomical variations are more prevalent among female patients (e.g., trochlear dysplasia, patella alta, and increased ligamentous laxity) and these conditions are known to alter patellofemoral mechanics, placing female patients at greater risk for instability.^26^ In addition, hormonal influences such as cyclic variations in estrogen and progesterone levels may impact tensile strength and elasticity of ligaments, which may influence injury risk and surgical outcomes. ^25^ Further studies are needed to clarify the extent of sex-based disparities and to determine whether targeted interventions, such as addressing anatomical variations or hormonal influences, can improve outcomes after MPFLR in female patients.

### Limitations

There are several limitations to this study. There were no randomized-controlled trials that examined the impact of race, insurance, or socioeconomic status, and only a small number of high-quality studies fulfilled the inclusion criteria. The majority of the studies were Level of Evidence III. These study designs are inherently prone to bias and confounding compared with greater levels of evidence. Most of the study designs limited the ability to identify the root cause of disparities in the evaluation and treatment of patients with patellar instability. In addition, the research designs made it more difficult to separate variables and determine how each one affects relevant outcomes (such as excluding those with poor socioeconomic status from having public insurance). Moreover, the study by Martinazzi et al.^21^ focuses specifically on female patients and the risk of patellar instability in relation to hormonal contraceptive therapy, which accounts a majority of the total population in this systematic review. Including a single large cross-sectional study that constitutes such a high proportion of the total sample may disproportionately influence the findings and limit their generalizability. It is important to note that all patients were within the United States, a country with a mixed health care system that includes both commercial insurance and public insurance programs such as Medicaid. This distinction is essential, as access to care, diagnostic capabilities, and treatment options can vary significantly between healthcare systems and socioeconomic contexts. Lastly, the decision to proceed with MPFLR also may be influenced by contextual factors not captured in a purely clinical context, i.e., logistical challenges like transportation that complicate postoperative rehabilitation and the ability of patients to take sufficient recovery time off of work.

## Conclusions

This systematic review found that lower socioeconomic status, public insurance coverage, and minority racial or ethnic identity were associated with delayed evaluation, lower likelihood of surgical intervention, and reduced postoperative compliance or satisfaction among patients undergoing MPFLR. Patients with these characteristics experience longer wait times from injury to clinic evaluation and have reduced odds in selection as a candidate for surgery. Postoperative satisfaction was also markedly worse for surgical patients with these characteristics.

## Disclosures

All authors (E.L.B., K.T.N., D.P.D., L.A.W., S.M.S., S.M., M.K.M.) declare that they have no known competing financial interests or personal relationships that could have appeared to influence the work reported in this paper.
