# Supplementary material for: Insurance Status and Income Proxies Are the Most Consistent Predictors of Disparities in Access to Care and Outcomes After Medial Patellofemoral Ligament Reconstruction in the United States: A Systematic Review
Source: Arthrosc Sports Med Rehabil. 2025 Sep 25;7(6):101268. doi: 10.1016/j.asmr.2025.101268 (PMC12800792; doi:10.1016/j.asmr.2025.101268)
Supplement: Appendix (1) [file mmc1.docx]

**Appendix:**

| **Database** | **Search Strategy** |
| --- | --- |
| **Pubmed** | **((((((Patellar instability[Title/Abstract]) OR (patellar reconstruction[Title/Abstract])) OR (medial patellofemoral ligament reconstruction[Title/Abstract])) OR (MPFL[Title/Abstract])))) AND (((((((("Health Services Accessibility"[Mesh]) OR "Insurance"[Mesh]) OR "Racial Groups"[Mesh]) OR "Race Factors"[Mesh]) OR "Socioeconomic Factors"[Mesh]) OR (((((((access to care[Title/Abstract]) OR (healthcare access[Title/Abstract])) OR (insurance[Title/Abstract])) OR (race[Title/Abstract])) OR (economics[Title/Abstract])) OR (socioeconomic status[Title/Abstract])) OR (socioeconomic factors[Title/Abstract])))) OR (("Sex Characteristics"[Mesh]) OR ((((sex differences[Title/Abstract]) OR (gender differences[Title/Abstract])) OR (sex characteristics[Title/Abstract])) OR (gender characteristics[Title/Abstract]))))** |
| **Embase** | ('patellar instability':ti,ab OR 'patellar reconstruction':ti,ab OR 'medial patellofemoral ligament reconstruction':ti,ab OR 'mpfl':ti,ab) AND ('health care access'/exp OR 'insurance'/exp OR 'ancestry group'/exp OR 'race'/exp OR 'socioeconomics'/exp OR 'sex'/exp OR 'health services accessibility':ti,ab OR 'racial groups':ti,ab OR 'race factors':ti,ab OR 'access to care':ti,ab OR 'healthcare access':ti,ab OR 'insurance':ti,ab OR 'race':ti,ab OR 'economics':ti,ab OR 'socioeconomic status':ti,ab OR 'socioeconomic factors':ti,ab OR 'sex differences':ti,ab OR 'gender differences':ti,ab OR 'sex characteristics':ti,ab OR 'gender characteristics':ti,ab) |
| **Cinahl** | (TI ( "patellar instability" or "patellar reconstruction" or "medial patellofemoral ligament reconstruction" or "MPFL" ) OR AB ( "patellar instability" or "patellar reconstruction" or "medial patellofemoral ligament reconstruction" or "MPFL" ) ) AND (( (MH "Health Services Accessibility") OR (MH "Insurance") OR (MH "Race Factors") OR (MH "Socioeconomic Factors") OR (MH "Sex Factors") ) OR TI ( "Health Services Accessibility" or "Insurance" or "Racial Groups" or "Race Factors" OR "Socioeconomic Factors" or "access to care" or "healthcare access" or "insurance" or "race" or "economics" or "socioeconomic status" or "socioeconomic factors" or "Sex Characteristics" or "sex differences" or "gender differences" or "sex characteristics" or "gender characteristics" ) OR AB ( "Health Services Accessibility" or "Insurance" or "Racial Groups" or "Race Factors" OR "Socioeconomic Factors" or "access to care" or "healthcare access" or "insurance" or "race" or "economics" or "socioeconomic status" or "socioeconomic factors" or "Sex Characteristics" or "sex differences" or "gender differences" or "sex characteristics" or "gender characteristics" )) |
| **Web of Science** | ("patellar instability" or "patellar reconstruction" or "medial patellofemoral ligament reconstruction" or "MPFL" (Title) or "patellar instability" or "patellar reconstruction" or "medial patellofemoral ligament reconstruction" or "MPFL" (Abstract)) **AND ("Health Services Accessibility" or "Insurance" or "Racial Groups" or "Race Factors" OR "Socioeconomic Factors" or "access to care" or "healthcare access" or "insurance" or "race" or "economics" or "socioeconomic status" or "socioeconomic factors" or "Sex Characteristics" or "sex differences" or "gender differences" or "sex characteristics" or "gender characteristics"** (Title) or **"Health Services Accessibility" or "Insurance" or "Racial Groups" or "Race Factors" OR "Socioeconomic Factors" or "access to care" or "healthcare access" or "insurance" or "race" or "economics" or "socioeconomic status" or "socioeconomic factors" or "Sex Characteristics" or "sex differences" or "gender differences" or "sex characteristics" or "gender characteristics"**(Abstract)) |
